# Characterization of Forage Quality, Phenolic Profiles, and Antioxidant Activity in Alfalfa (*Medicago sativa* L.)

**DOI:** 10.3390/plants11202735

**Published:** 2022-10-16

**Authors:** Daniela Horvat, Marija Viljevac Vuletić, Luka Andrić, Renata Baličević, Marija Kovačević Babić, Marijana Tucak

**Affiliations:** 1 Agricultural Institute Osijek, Južno predgrađe 17, 31000 Osijek, Croatia; 2 Faculty of Agrobiotechnical Sciences Osijek, Josip Juraj Strossmayer University of Osijek, Vladimira Preloga 1, 31000 Osijek, Croatia

**Keywords:** alfalfa, antioxidants, HPLC, nutrition, polyphenols

## Abstract

Alfalfa (*Medicago sativa* L.) is one of the most important forage species and is recently more in focus for human consumption mainly due to its content of bioactive phenolics. Samples of the seventeen alfalfa cultivars/populations were collected at the Agricultural Institute Osijek with the aim to evaluate their forage quality, phenolic profiles, and antioxidant potential. Significant differences (*p* < 0.05) existed among studied alfalfa in all analyzed traits. The cultivar OS 99 and populations L7 and L20 were characterized by high crude protein content (22.5–24.7%) and the lowest neutral (40.2–42.9%) and acid detergent fibres (33–35.5%). The soluble-free phenolics from alfalfa leaves were extracted by methanol while insoluble cell-wall bound phenolics were released by alkaline hydrolysis. The bound phenolic extract showed a stronger DPPH scavenging capacity (20.8 mg TE/g dm) than the soluble (11.4 mg TE/g dm). The HPLC data revealed that more phenolics were found in the bound (3638.0 μg/g dm) than in the soluble form (912.3 μg/g dm). In the soluble extract of the alfalfa leaves, the major compound was catechin (338.3 μg/g dm), while rutin, epicatechin, and ferulic acid were minor ones. In the bound phenolic extract, the most abundant was ferulic (2198.2 μg/g dm) and *p*-coumaric acid (983.7 μg/g dm), followed by myricetin, apigenin, and quercetin. The principal component analysis revealed that alfalfa cultivars/populations were better discriminated based on the data on phenolics, rather than on forage quality. The cultivars/populations Florida 66, OS 66, L 40, L 42, Seed Force 4, and Torlesse were the most interesting in terms of phenolic health-promoting characteristics.

## 1. Introduction

Alfalfa (*Medicago sativa* L.) is a perennial herb belonging to the Fabaceae family. Globally, it is one of the most important forage crops due to its high biomass production and good nutritive quality [1]. The quality characteristics of forage legumes have a very complex nature mostly influenced by maturity (harvest date), storage, environment (moisture, temperature, sunlight amount), trial field management, soil fertility, and cultivar [2,3,4,5,6,7].

Recently, alfalfa has been proposed as an important source of protein for human nutrition due to its high protein content and could therefore be a good inexpensive and alternative animal protein source [8]. Alfalfa is a crop that provides a higher yield of proteins per unit area than any field crop, but apart from that, increasing attention has been paid to the presence of non-nutritional bioactive components in alfalfa with health-promoting effects. Numerous authors have reported that alfalfa contains bioactive phytochemicals such as alkaloids, saponins, phenols, tannins, polysaccharides, and phytoestrogens, with antioxidant anti-inflammatory, immunostimulatory, and anticarcinogenic properties [9,10,11].

In addition to the extensive research of Medicago species triterpene saponins and phytoestrogens [12,13,14], alfalfa contains potentially valuable phenolics with an excellent ability to capture oxidative free radicals [12,15]. Several classes of alfalfa phenolics were reported in the literature, and among them, phenolic acids and flavonoids are widely present [9,16,17].

Alfalfa is recently in use as a raw material to produce products for human consumption; however, its nutraceutical and pharmaceutical potential has not been assessed. Besides its nutritional composition, it is advisable to evaluate the antioxidant capacity and phenolic content in new alfalfa-derived products [18]. Phenolics, according to their existing forms in plants, are generally presented as soluble-free, soluble-esterified that are conjugated to sugars and low-molecular-mass components and insoluble-bound, which are covalently bound to the cell-wall structural components such as cellulose, hemicellulose, or lignin [19,20,21]. In vivo, the bound phenolics are released by colonic microflora within the gastrointestinal tract. Girish et al. [22] showed that the insoluble phenolics from black gram (*Vigna mungo*), when released, have the same health benefits as soluble phenolics considering the same positive antioxidant effect.

Most of the studies in alfalfa leaves have concerned the total phenols and extractable soluble-free phenolics on an individual level, while insoluble-bound ones have been neglected. To the best of our knowledge, such a comprehensive characterization of targeted phenolics has not been conducted in alfalfa cultivars and research populations grown in Croatia. Therefore, the current study evaluated the forage quality, the profiles of both soluble and bound phenolics, as well as their antioxidant activity in seventeen domestic and foreign alfalfa cultivars/populations grown at the Agricultural Institute Osijek. The main objectives were to select the most promising alfalfa materials as a potential source of phenolic compounds, because of their possible use in the future in our forage crop breeding programme and/or for nutraceutical purposes. Moreover, the differences among studied alfalfa for all analyzed traits were assessed.

## 2. Results and Discussion

### 2.1. Nutritive Forage Quality of Alfalfa

Analysis of variance demonstrated statistically significant differences in all of the quality traits studied (*p* < 0.05) (Table 1 and Table S1). Alfalfa leaves are rich in highly digestible proteins; however, so far, they have not been fully utilized in the human diet. Across cultivars/populations, the crude protein (CP) ranged from 20.4% (L 43) to 24.7% (OS 99).

In contrast to CP, fibre content is inversely related to forage quality [23]. Crude fibre content (CF) varied from 27.9% (OS 200) to 35.7% (L 43) (Table 1 and Table S1). Acid detergent fibre (ADF) and neutral detergent fibre (NDF) represent highly indigestible and partially digestible fibre portion of forage and their higher content reduces the energy value of alfalfa for feeding livestock [24]. L 20 and OS 100 had the lowest NDF values (40.2–40.8%), while the lowest ADF (33.0%) was also present in L 20. The digestibility and utilization of alfalfa by livestock is also hampered by lignin content [25,26]. The lowest lignin (ADL) was noticed in OS 99 (6.9%), followed by OS 100, Seed Force 4, OS 88, and Florida 88 (ADL below 8%) (Table 1 and Table S1). According to the Hay Marketing Task Force of the American Forage and Grassland Council USDA [27], on average, studied alfalfa related to CP (22.2%), NDF (43.7%), and ADF (38.4%) obtained prime, premium, and good forage quality, respectively (Table 1 and Table S1). In the literature, a wide range of CP and fibre content in alfalfa has been observed due to the influence of many factors, such as cultivar, climate, agronomic practices, and their interactions [14,23]. Scholtz et al. [28] analyzed the forage quality of 168 alfalfa hay samples and reported a large variation for CP (13.9–27.8%), NDF (28.9–65.9%), and ADF (21.3–47.3%).

### 2.2. Total Phenolic Content (TPC) and Antioxidant Activity (AOA) of Alfalfa

**Figure 1:**
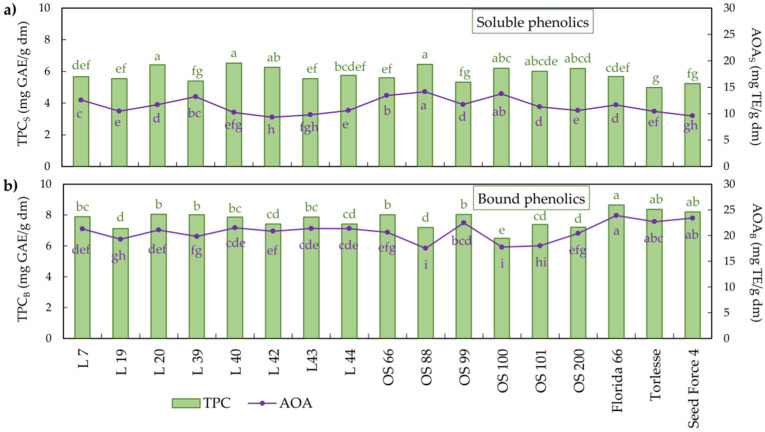
Mean values of TPC and AOA of soluble (**a**) and bound (**b**) phenolic extracts of alfalfa. Different letters indicate significant difference among accessions at *p* < 0.05 according to the LSD test. TPC—total phenolic content; AOA—antioxidant activity of soluble (_S_) and bound (_B_) phenolics.

Plant phenolics can serve as antioxidants through different reaction pathways, such as inhibition of lipid peroxidation, metal chelation, quenching of singlet oxygen, and radical scavenging [29]. The Folin–Ciocalteu assay is widely used to estimate TPC in plants despite some nonphenolic interfering substances [30]. Bound phenolics of alfalfa leaves compared to soluble ones showed higher antioxidant capacity in terms of TPC (7.7 and 5.8 mg GAE/g dm, respectively) and AOA (20.8 and 11.4 mg TE/g dm), respectively (Figure 1; Table S2).

Many factors, such as solid–liquid ratio, extraction time and temperature, pH, solvent composition, and particle size, could markedly impact the TPC and their AOA [31]. Moreover, some authors have observed genotype, location, and harvesting time effects on these traits [32,33]. Among studied alfalfa cultivars/populations, statistically significant differences in TPC and AOA were noted (Figure 1; Table S2). Torlesse had the lowest soluble TPC, followed by lower AOA (5.0 mg GAE/g dm and 10.4 mg TE/g dm), while L 20, OS 88, and L 40 had the highest TPC (6.4–6.5 mg GAE/g dm) with inconsistent AOA ranging from 10.2 to 14.2 mg TE/g dm. OS 100 had the lowest bound TPC (6.5 mg GAE/g dm) and the lowest AOA, as well as OS 88 (17.7 and 17.5 mg TE/g dm), respectively. Florida 66 had the highest TPC and AOA (8.6 mg GAE/g dm and 23.9 mg TE/g dm, respectively). TPC contents in soluble extracts revealed in our study are in accordance with the findings of previous reports, which also determined TPC in alfalfa leaves in 80% methanol extract [31,32,33] but they were mainly focused on TPC of soluble phenolics.

### 2.3. Phenolic Compounds in Alfalfa

In contrast to the extensively studied triterpene saponins and the phytoestrogen compounds of *Medicago* species [5,12,13], *Medicago* polyphenols are less genus-specific and generally encountered in many legumes [34]. As we mentioned before, most plant phenolics are present in soluble and insoluble-bound forms.

**Figure 2:**
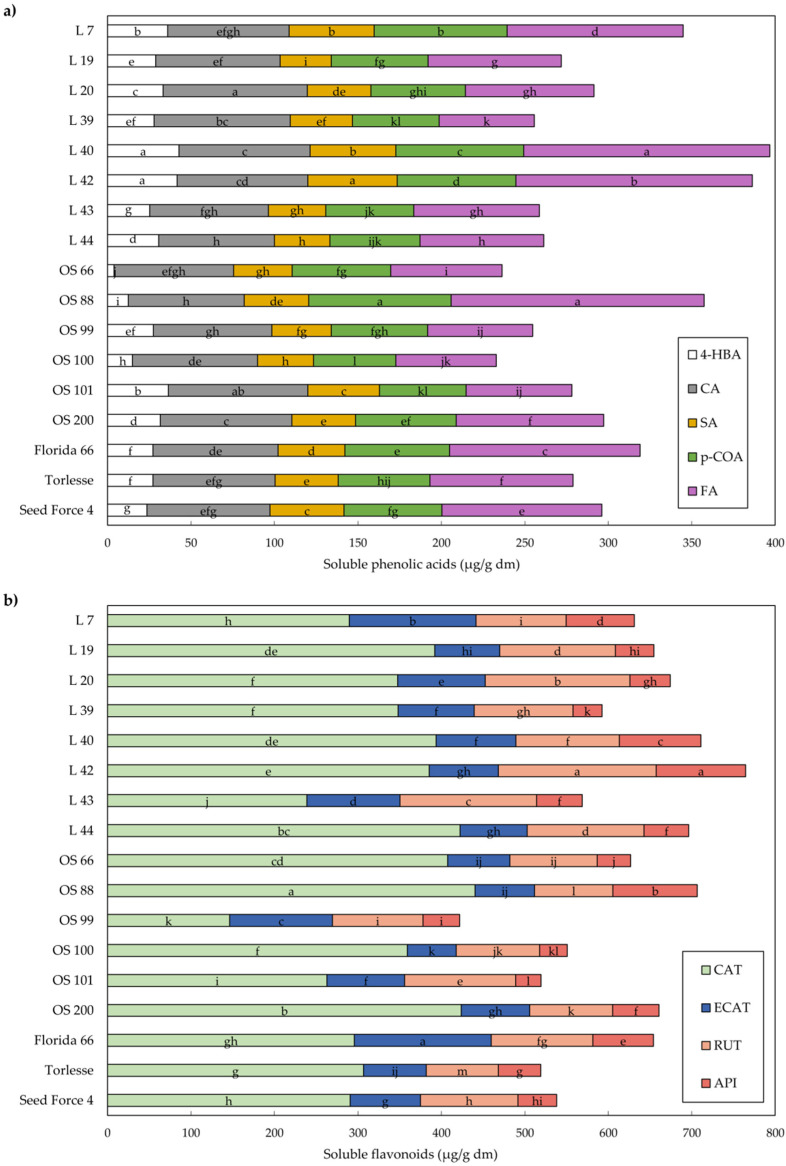
Mean values of soluble phenolic acids (**a**) and flavonoids (**b**) in alfalfa. Different letters indicate significant difference among accessions at *p* < 0.05 according to the LSD test. 4-HBA—4-hydroxybenzoic; CAT—catechin; CA—caffeic acid; SA—syringic acid; ECAT—epicatechin; p-COA—p-coumaric acid; FA—ferulic acid; RUT—rutin; API—apigenin.

**Figure 3:**
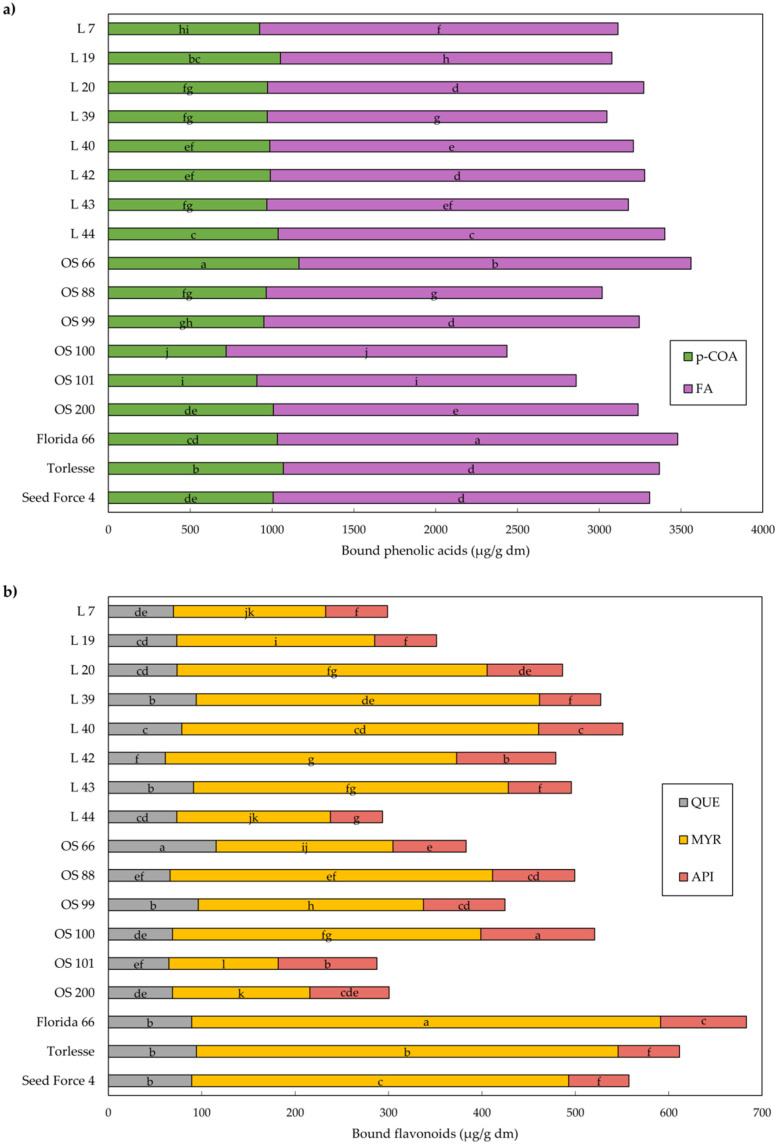
Mean values of bound phenolic acids (**a**) and flavonoids (**b**) in alfalfa. Different letters indicate significant difference among accessions at *p* < 0.05 according to the LSD test. p-COA—p-coumaric acid; FA—ferulic acid; QUE—quercetin; MYR—myricetin; API—apigenin.

Soluble phenolics can be easily directly extracted using a range of solvents while for bound phenolics extraction, alkaline hydrolysis was found to be the most efficient method [19,29]. Our HPLC-DAD analysis, which was set up for the detection and quantification of 18 phenolics, revealed the presence of 11 phenolics. The total amount of bound phenolics in alfalfa leaves (3638.0 μg/g dm) was markedly greater compared to soluble ones (912.3 μg/g dm) (Figure 2 and Figure 3; Tables S3 and S4).

The HPLC results showed that the content and composition of phenolics in alfalfa leaves depended on cultivars/populations; hence, followed by the total phenolics (TP), in soluble extract, TP ranged from 676.7 μg/g dm (OS 99) to 1150.9 μg/g dm (L 42) (Figure 2). The lowest content of bound phenolics was found in OS 100 (2957.2 ug/g dm), while Florida 66 had the highest content (4163.6 μg/g dm) (Figure 3; Table S4).

With regard to phenolic acids in alfalfa leaves (Figure 2a and Figure 3a), FA was the most abundant in both soluble and bound extract (91.0 μg/g dm and 2198.2 μg/g dm), respectively. FA and p-COA were present in both soluble and bound forms, while 4-HBA, CA, and SA acids were present only in soluble form. Statistical analysis of the identified phenolic acids revealed a wide range of content differences among samples. In the bound extract, dominant FA and p-COA accounted for 87.5% of the total identified phenolic acids. Foreign cultivar Florida 66 had 42.5% more FA than the OS 100 (1717.6 μg/g dm), while Croatian cultivar OS 66 and population L 44 had 39.5% and 37.7%, respectively, higher content of FA compared to OS 100. The difference between the highest p-COA content in OS 66 (1164.5 μg/g dm) and the lowest in OS 100 (718.9 μg/g dm) was 62.0% (Figure 3a; Table S4).

The levels of phenolic acids in alfalfa leaves reported by others varied in a broad range because their compositions and concentrations depended on several factors such as extraction type (acid, alkaline, ultra-sound, microwave, supercritical CO2, etc.), used analytical technique (HPLC, HPLC-MS, HPLC-MS/MS, etc.), and alfalfa part (aerial or roots) [16,35,36]. Newby et al. [37] determined the dominance of alkali-labile bound phenolic acids in relation to soluble ones (less than 2%). These authors found 4-HBA, VA, p-COA, and FA, both soluble and bound, while salicylic and sinapic acids occurred only as bound. Bohn and Fales [38], in alfalfa samples pre-treated with ethanol, recorded content of p-COA (900.0 μg/g dm) and FA (1890.0 μg/g dm) similar to ours. In the alfalfa leaves, Bajkacz et al. [16] noted 3-(4-hydroxyphenyl) propionic, CA, 4-HBA, and BA as the major phenolic acids but in very low concentrations (from 632.8 to 10.000 ng/g), while Igual et al. [15] noted 2-HB as the dominant one (1440.64 μg/g dm). Hydroxycinnamic acids, especially FA and p-COA, are under increasing attention because of their association with plant cell wall lignification [29]. Many studies have addressed the relationships of ester and/or ether-linked FA with rumen nutrient digestibility, and there is still some controversy about whether these linkages could be used as a predictor of forage digestibility in ruminants [39].

Furthermore, in our study, the HPLC analysis revealed that flavonoids CAT, ECAT, and RUT were identified only in the soluble extract of alfalfa leaves, while QUE and MYR only in the bound. API was present in both extracts but it was more abundant in the extract of bound phenolics (Figure 2b and Figure 3b). The highest content of CAT, as the dominant soluble flavonoid, had Croatian OS 66, L 44, OS 200, and OS 88 (407.4–440.3 μg/g dm), which means that these samples had several times more CAT content than the lowest one (146.4 μg/g dm in OS 99). The highest MYR, as the most abundant flavonoid in bound extract, had foreign cultivar Florida 66 (502.2 μg/g dm), which is 4.3 times more than the lowest value in OS 101 (117.1 μg/g dm) (Figure 3b; Table S4). In alfalfa samples, in addition to the flavonoids found in our research, some authors also mentioned glabridin, naringin, and luteolin as major flavonoids [13,36,40,41,42].

### 2.4. Principal Component Analysis

**Figure 4:**
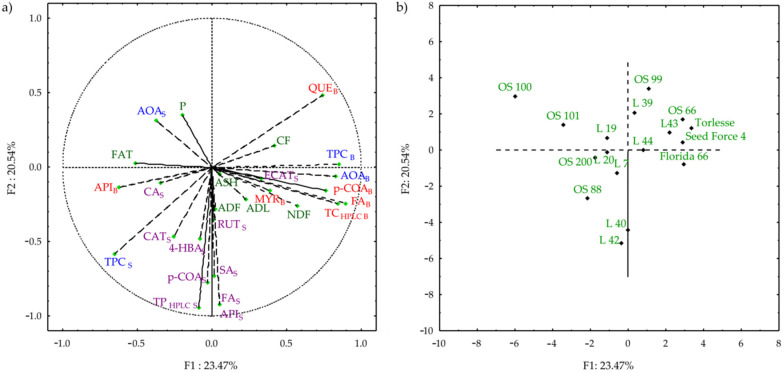
Biplot relative to the principal component analysis (factor loadings (**a**) and scores (**b**) of first two factors) performed on the nutritive and data on phenolics (CP—crude protein; FAT—crude fat; CF—crude fibre; NDF—neutral detergent fibre; ADF—acid detergent fibre; ADL—acid detergent lignin. TPC—total phenolic content; AOA—antioxidant activity; 4-HBA—4-hydroxybenzoic; CAT—catechin; CA—caffeic acid; SA—syringic acid; ECAT—epicatechin; p-COA—p-coumaric acid; FA—ferulic acid; RUT—rutin; QUE—quercetin; MYR—myricetin; API—apigenin; TP_HPLC_—total phenolics obtained by HPLC; _S_—soluble phenolics; _B_—bound phenolics).

Principal component analysis (PCA) was performed to obtain better insight into the data and to understand the relationships between variables and samples. Based on the PCA results performed from the correlation matrix (Table S5), the first eight PCs were extracted with an eigenvalue greater than 1, and together they explained 88.9% of the total variance. The PC1 accounted for 23.47%, and PC2 for 20.54% of the variance in the dataset (Figure 4; Table S6). According to the loaded values, the total bound phenolics, including FA, and p-COA, and their AOA, together with NDF and QUE determined sample distribution along PC1, whereas soluble phenolics together with soluble FA, p-COA, SA, and API determined sample distribution along PC2 (Figure 4; Tables S7 and S8).

The PCA analysis illustrated that the AOA of the bound extracts was mainly attributed to the phenolics, which is confirmed by their strong correlation (r = 0.822, Table S5). This observation is in agreement with others [31,35]. The distance between TPC and their AOA in soluble extract indicates those other compounds acting as antioxidants, rather than the identified phenolics [29]. The obtained weak correlation between TPC and AOA in the soluble extract (r = 0.163, Table S5) was also reported by Soto-Zarazúa et al. [18]. In our study, the PCA revealed that alfalfa cultivars and populations were better discriminated based on the data on phenolics, rather than on forage quality. OS 66, L 43, Torlesse, and Seed Force were strongly characterized by the bound TPC, QUE, and CF. Florida 66 is slightly separated from the previously mentioned materials due to the highest bound MYR, FA, and NDF. OS 99 and L 39 are gathered around QUE_B_ and CF, while OS 100 around CP and AOA_S_. L 42, L 40, and OS 88 are the most characterized by soluble FA, SA, p-COA, 4-HBA, and API. Other cultivars/populations were grouped around the centre, which was more characterized by forage quality traits.

## 3. Materials and Methods

### 3.1. Alfalfa Cultivation and Sampling

A field trial with seventeen alfalfa (*Medicago sativa* L.) materials was established on 7 March 2019 at the Agricultural Institute Osijek (45°32′ N and 18°44′ E, altitude 90 m). Alfalfa materials included six domestic (OS 66, OS 88, OS 99, OS 100, OS 101, OS 200), three foreign cultivars (Florida 66, Torlesse, Seed Force 4), and eight research populations (L 7, L 19, L 20, L 39, L 40, L 42, L 43, L 44). Seed samples of foreign cultivars were obtained from the Margot Forde Forage Germplasm Centre New Zealand’s seed bank for perennial grasses and legumes. Second-cutting alfalfa was harvested in 2020 at the late bud to early flowering stage of development. For the analysis of forage quality average bulk green mass samples were taken (approximately 500 g), dried at 60 °C for 48 h and ground with an ultra-centrifugal mill with a 1 mm diameter sieve (Retsch Type ZM1, Haan, Germany). For the analysis of phenolics and their antioxidant activity, the average leaf samples (healthy, young, and fully developed ones) were randomly collected from the plants in the middle of each plot, just before second-cutting. The collected leaves were stored in a refrigerator (−80 °C), lyophilized, and ground into a fine powder by an oscillating mill immediately before extraction.

### 3.2. Chemicals

Chemical materials used in this study were of analytical or HPLC-grade. Purified water from a Milli-Q Element A10 System (Millipore, Milford, MA, USA) was used for the sample, reagent solutions, and mobile phase preparation. According to previously reported data of *Medicago sativa* L. phenolic compounds, the next phenolic acids and flavonoids purchased from Sigma-Aldrich (Saint Louis, MO, USA) were analyzed: gallic acid (GAL), chlorogenic acid (CHA), vanillic acid (VA), caffeic acid (CA), syringic acid (SA), sinapic acid (SIA), p-coumaric acid (p-COA), ferulic acid (FA), 3-hydroxybenzoic acid (3-HBA), benzoic acid (BA), 3,4-dihydroxybenzoic acid (3,4-DHBA), p-hydroxybenzoic acid (p-HBA), catechin (CAT), epicatechin (EPI), rutin (RUT), quercetin (QUE), naringenin (NAR), and keampferol (KEA). Standard stock solutions (1.0 mg/mL) of phenolic acids and flavonoids were prepared by dissolving them in methanol.

### 3.3. Nutritive Forage Analysis

Crude proteins (CP) were determined by the Kjeldahl method (FOSS Kjeltec 2300, Hoganas, Sweden). Crude fibre (CF), acid and neutral detergent fibre and acid detergent lignin (ADF, NDF, ADL, respectively) content expressed in dry matter (dm) was measured according to the standard protocol using FIWE6 Raw Fiber Extractor (Velp Scientifica, Usmate, Italy). Ash content was measured by combusting a known amount of leaves for 6 h at 550 °C, and crude fat (FAT) was extracted by the Soxhlet method.

### 3.4. Extraction of Soluble and Bound Phenolics

The soluble and bound phenolics were extracted according to Zavala-López and García-Lara [43] with slight modifications. For soluble phenolics, 80% methanol was used as the extraction solvent; 3.5 mL were added to 250 mg of the alfalfa lyophilized leaves and the mixture was homogenized for 2 min at 2500 rpm on a vortex (MSV-3500 Biosan, Riga, Latvia). The sample was incubated at 25 °C for 15 min at room temperature and constant agitation of 500 rpm (Shaking water baths GFL 1092, Burgwedel, Germany), followed by centrifugation (Universal 320R, Hettich, Tuttlingen, Germany) for 10 min at 5000 rpm. The supernatant was decanted and stored at −20 °C until analysis. Sample extraction of soluble phenolics was done in duplicate and used for further analysis. After removing soluble phenolics, the bound phenolics were extracted from the pellet residue with 2.5 mL of 2 M NaOH. The alkaline hydrolysis was conducted at 90 °C for 2 h (Shaking water baths GFL 1092, Burgwedel, Germany). After hydrolysis, the sample was acidified with 2.5 mL of 2 M HCl at pH 2. Lipids were removed by the addition of 4.0 mL of n-hexane. The bound phenolics were recovered three times by 4.0 mL of ethyl acetate. The collected ethyl acetate layer was evaporated to dryness (BÜCHI B-720 Vacuum Controller, Flawil, Germany) and resuspended in 3 mL of 80% methanol and stored at −20 °C until analysis. Sample extraction was done in duplicate and used for further analysis.

### 3.5. Total Phenolic Content (TPC)

Total phenolic content in soluble and bounded phenolics extracts was determined by the modified Folin–Ciocalteu method [44]. In brief, 50 μL of soluble and bound phenolic extract (1:1 diluted with 80% methanol) was mixed with 1.55 mL of dH_2_O and 0.1 mL of Folin–Ciocalteu reagent (1:1; *v*/*v* diluted with dH_2_O). After 5 min, 0.3 mL of 20% Na_2_CO_3_ solution was added. The homogenized reaction mixture was left to stand for 60 min in a dark place at room temperature, after which absorbance readings were at 765 nm (Specord 200, Analytik Jenna GmbH, Jena, Germany) against methanol as a blank. The content of total polyphenols was expressed as mg of gallic acid equivalents (GAE) per g of dm based on a gallic acid calibration curve. All measurements were performed in duplicate.

### 3.6. Antioxidant Activity (AOA)

The antioxidant activity of soluble and bound phenolics was analyzed using the 2,2-diphenyl-1-picrylhydrazyl (DPPH) assay [45]. Briefly, a 0.5 mM solution of methanolic DPPH solution was prepared. The initial absorbance of the DPPH in methanol (control) was measured at 517 nm and did not change throughout assay. 0.2 mL of each sample (1:5 diluted with 80% methanol) was mixed with 2 mL of methanol and 1 mL of methanolic DPPH solution. Discolourations were measured at 517 nm (Specord 200, Analytik Jenna GmbH, Jena, Germany) after incubation for 30 min at room temperature in the dark, and the data were presented as mg of the Trolox equivalent (TE) per g of dm based on a Trolox calibration curve. All measurements were performed in duplicate.

### 3.7. Determination and Quantitation of Phenolics by HPLC

Individual phenolics in soluble and bound extracts were analyzed using a Series 200 HPLC system (Perkin Elmer, Waltham, MA, USA) coupled with a Kinetex Core-Shell RP-C18 column (150 × 4.6 mm, 100 Å, 5 μm) and A diode array detector (DAD). Prior to HPLC analysis samples were filtered through a 0.2 μm nylon filter (Ahlstrom GmbH, Helsinki, Finland). The mobile phase for analysis included solvent A (Millipore water acidified with 1% trifluoroacetic acid (*v*/*v*)) and solvent B (acetonitrile acidified with 1% trifluoroacetic acid (*v*/*v*)). Elution was performed using linear gradients from 5−40% B in 40 min, isocratic 90% of B for 5 min and column equilibration in 5 min. At a column temperature of 30 °C and a flow rate of 1.0 mL/min, peaks were detected at 275 nm. Phenolic compounds were identified by comparison of UV absorption spectra and retention times with those of standards, while its quantification was done using a five-point external calibration curve.

### 3.8. Statistical Analysis

Differences in measured variables of alfalfa cultivars were evaluated using analysis of variance (ANOVA) followed by the post hoc Least Significant Difference (LSD) test. Differences were considered significant if *p* < 0.05 and indicated by different letters. Data presented in the text, tables, and figures are mean values of two replicates (*n* = 2). Correlations among the measured parameters and alfalfa cultivars were explored by Principal Component Analysis (PCA). PCA was performed using the correlation matrix of the average values of traits. Linear correlations among variables were determined by Pearson coefficients (*p* < 0.05).

## 4. Conclusions

Significant differences were found among alfalfa cultivars/populations in forage quality, phenolic profiles, and antioxidant potential. The principal component analysis revealed that alfalfa cultivars/populations were better discriminated based on the data on phenolics, rather than on forage quality. The obtained results pointed out L 7, L 20, and OS 99 as Croatian cultivars/populations with the best forage quality, while Florida 66, OS 66, L 40, L 42, Seed Force 4, and Torlesse were the most interesting in terms of the phenolic health-promoting characteristics. Investigation of these cultivars/populations over a long period of time is necessary to fully understand their superior traits, because of their possible use for breeding and/or nutraceutical purposes.

## Figures and Tables

**Table 1:** Mean values of forage nutritive quality traits in alfalfa expressed in dry matter (dm).

Cultivar/Population	CP (%)	ASH (%)	FAT (%)	CF (%)	NDF (%)	ADF (%)	ADL (%)
L 7	23.0 bc	11.2 cd	1.8 bcd	30.7 i	42.6 h	35.5 ij	8.0 cde
L 19	21.7 ef	11.6 a	2.1 a	31.9 fgh	43.9 ef	37.4 gh	8.7 bc
L 20	22.5 cd	11.5 ab	1.9 bcd	29.1 j	40.2 i	33.0 k	8.0 cde
L 39	23.0 bc	11.2 cd	1.7 de	33.3 cde	44.3 cde	39.5 cde	8.8 bc
L 40	22.2 de	11.4 b	1.6 efg	28.9 jk	44.7 bcd	39.7 cde	8.7 bc
L 42	20.9 gh	10.8 gh	1.5 fg	32.8 cdef	44.3 cdef	41.7 a	8.7 bc
L 43	20.4 h	11.0 ef	1.4 g	35.7 a	43.6 efg	39.3 def	9.4 ab
L 44	21.3 fg	10.6 i	1.7 cde	32.6 def	43.5 fg	38.0 g	9.7 a
OS 66	21.7 ef	10.7 hi	1.1 h	33.7 bc	45.5 b	41.6 ab	8.4 cde
OS 88	21.4 f	10.9 fg	1.8 bcd	33.6 bcd	45.0 bc	40.7 abc	7.7 ef
OS 99	24.7 a	11.0 f	1.5 efg	30.6 i	42.9 gh	34.7 j	6.9 f
OS 100	22.3 d	10.8 gh	2.0 ab	31.2 hi	40.8 i	38.0 fg	7.6 ef
OS 101	23.1 b	11.1 de	1.7 def	31.0 hi	42.4 h	40.4 bcd	8.3 cde
OS 200	23.3 b	10.4 j	1.7 de	27.9 k	44.0 def	38.6 efg	8.0 cde
Florida 66	23.1 b	11.2 cd	1.7 bcde	31.4 ghi	43.6 efg	36.6 hi	7.8 def
Torlesse	21.5 f	11.3 c	1.4 g	32.4 efg	43.9 def	39.3 def	8.6 bcd
Seed Force 4	21.4 f	10.8 gh	1.9 abc	34.5 b	47.2 a	38.0 g	7.7 ef

Different letters indicate significant difference among accessions at *p* < 0.05 according to the LSD test. CP—crude protein; FAT—crude fat; CF—crude fibre; NDF—neutral detergent fibre; ADF—acid detergent fibre; ADL—acid detergent lignin.

## Data Availability

Not applicable.

## Supplementary Materials

The following supporting information can be downloaded at: https://www.mdpi.com/article/10.3390/plants11202735/s1, Table S1: Mean values ± standard deviation of forage nutritive quality traits in alfalfa expressed in dry matter (dm); Table S2: Mean values ± standard deviation of TPC and AOA of soluble and bound phenolics extracts of alfalfa; Table S3: Mean values ± standard deviation of soluble phenolics in alfalfa. 4-HBA—4-hydroxybenzoic; CAT—catechin; CA—caffeic acid; SA—syringic acid; ECAT—epicatechin; p-COA—p-coumaric acid; FA—ferulic acid; RUT—rutin; API—apigenin; Table S4: Mean values ± standard deviation of bound phenolics in alfalfa. P-COA—p-coumaric acid; FA—ferulic acid; QUE—quercetin; MYR—myricetin; API—apigenin; Table S5: Correlation matrix (Pearson (*p* < 0.05)) of traits measured in alfalfa; Table S6: Eigenvalues; Table S7: Factor loadings and correlations between variables and factors; Table S8: Factor scores.
